# Evaluation of the Michigan Clinical Consultation and Care Program: An Evidence-Based Approach to Perinatal Mental Healthcare

**DOI:** 10.3390/jcm12144836

**Published:** 2023-07-22

**Authors:** Maria Muzik, Rena A. Menke, Meriam Issa, Chelsea Fisk, Jordan Charles, Jennifer M. Jester

**Affiliations:** 1 Department of Psychiatry, University of Michigan-Michigan Medicine, Ann Arbor, MI 48109, USA; enaam@med.umich.edu (R.A.M.); meriss@med.umich.edu (M.I.); fiskc@med.umich.edu (C.F.); chajorda@med.umich.edu (J.C.); jjester@med.umich.edu (J.M.J.); 2 Department of Obstetrics & Gynecology, University of Michigan-Michigan Medicine, Ann Arbor, MI 48109, USA

**Keywords:** perinatal mental health, integrated care, psychiatry consultation, collaborative care, implementation science

## Abstract

Mood and anxiety disorders affect pregnant individuals and their families at increased rates throughout the perinatal period. Geographic, financial, and social barriers often preclude adequate diagnosis and treatment. The aim of this manuscript is to describe the consultation and care arms of the Michigan Clinical Consultation and Care (MC3) program, a statewide program designed to facilitate access to perinatal mental healthcare for OB/Gyn patients, and to describe the participants engaged in the program, examine the predictors of participant retention, and provide preliminary data regarding participants’ mental health outcomes. We enrolled 209 participants to the clinical care arm, of which 48 were lost to follow-up, while 107 remained enrolled at the time of data analysis. A total of 54 participants met their treatment goals. A total of 97% of participants asserted they were satisfied with the services they received. Black race and public insurance predicted faster attrition from the care arm treatment; risks for interpersonal violence exposure and substance use were unrelated to attrition. Preliminary mental health outcomes showed significant decreases in anxiety and depression, with the most dramatic decreases in the first month of treatment. Overall, the MC3 clinical care arm shows promising rates of adherence, excellent program satisfaction, and a positive impact on perinatal mental health, supporting continued program implementation and ongoing evaluation.

## 1. Introduction

### 1.1. Aims

The aim of this manuscript is to describe the consultation and care arms of the Michigan Clinical Consultation and Care (MC3) program, describe the participants who have been referred to the program, examine the predictors of participant retention in the care arm, and provide preliminary data regarding mental health outcomes of participants in the care arm. We will share early program implementation outcomes from since the program’s inception in 2021, including the number of participants served, participant demographics, the number and type of treatments delivered, participant satisfaction, retention rates in the program, and the preliminary findings on maternal mental health benefits.

### 1.2. Background

Mood and anxiety disorders affect pregnant individuals and their families at increased rates throughout the perinatal period. Perinatal depression affects between 11 and 15% of obstetric populations [1,2,3,4]. Individuals experience an onset of depressive episodes at the highest rate during the postpartum period (40.1%), pregnancy (33.4%), and before pregnancy (26.5%) [5]. A total of 19.3% of individuals across the peripartum period experience suicidal ideations [5], and suicidality in pregnancy and the postpartum period is on the rise in the US [6]. Anxiety affects up to 20% of individuals during pregnancy, and as many as 50% of individuals immediately postpartum [7]. Substance use occurs among at least 10% of perinatal individuals, and estimates hold that it accounts for 8–22% of maternal mortality [1]. Yet, despite these prevalence rates, prior work suggests that up to 70% of patients go undetected, approximately 85% go untreated, up to 93% are inadequately treated, and as many as 97% of patients continue to suffer from symptoms without remission [8,9].

Undertreated and underdiagnosed mental health concerns can impact families in a myriad of ways. A 2021 study found that people with perinatal mental health disorders experienced 50%-higher rates of severe maternal morbidity, and 87%-higher rates among those with trauma- or stress-related mental health disorders, as compared with people without perinatal mental health disorders [4]. This study also estimated an increased annual delivery cost of USD 102 million in the US among people with perinatal mental health conditions versus those without [4]. Mild and moderate symptoms may interfere with functioning and one’s ability to maintain jobs and households; severe symptoms may result in self-harm and even suicide or infanticide [8.9]. Moreover, mental illness can confer negative impacts on parenting and, in turn, infant emotional development and self-regulation, posing a risk to the mental health of the developing child [10,11,12].

Data from a recent Canadian study suggest increased rates of perinatal depression and anxiety during the COVID-19 pandemic, with more than a third of pregnant individuals reporting depression symptoms and more than half reporting anxiety symptoms [13,14]. Many healthcare institutions also saw increased rates of substance use and interpersonal violence (IPV) during this time, both of which precipitate additional risk for mental health concerns [15,16,17]. Prior to COVID-19, Black and Indigenous People and People of Color (BIPOC) already experienced increased risks for adverse mental health outcomes and faced structural barriers to accessing perinatal mental healthcare at baseline. The pandemic only compounded these existing inequities [18].

The vast majority of pregnant individuals in America interact with medical professionals over the course of their pregnancies. In 2016, 77.1% of pregnant individuals received obstetric care beginning in their first trimester of pregnancy [19]. Obstetrician–gynecologists (OB/GYNs) are, in many cases, the only healthcare provider that women of childbearing age see on a regular basis [20,21]. OB/GYN visits represent one-third of all office visits for women between the ages of 18 and 45, and most non-illness-related office visits for women under 65 years of age [20,21]. However, perinatal mental health symptoms are often undertreated due to a lack of training, staffing shortages, and time constraints within OB/GYN office settings, as well as the sociocultural stigma surrounding mental health conditions, which makes patients reticent to admit to symptoms [20]. Given the ubiquity of OB/GYN care, combined with the barriers to accessing mental health services, OB/GYN offices are an optimal site for addressing perinatal mental health through integrated care.

Integrating mental health into primary and obstetrical care can greatly improve mental health outcomes [20,21]. US obstetric trials have shown improved depression outcomes in pregnancy and the postpartum period with integrative collaborative care, including an engagement session, assessment by a member of the care team, a patient-led choice between an antidepressant medication or psychotherapy, and patient outreach following any missed appointments [3]. Integrating mental health screening within obstetric care aligns with the current recommendations from the American College of Obstetrics and Gynecologists, which recommends screening for depression, bipolar disorder, anxiety, trauma, IPV, adverse childhood experiences, substance use disorders, and social determinants of health [1,22]. Other professional organizations, including both the American Psychiatric Association and the US Preventive Services Task Force, recommend mental health screening as well [1]. Interventions, like the Massachusetts Child Psychiatry Access Program (MCPAP) for Moms, expand screening for pregnant individuals among obstetric providers, building capacity both at the provider and practice levels to promote appropriate treatment and diagnosis [18,23].

One of the models of integrated behavioral healthcare is the Collaborative Care (CoCM) model. As part of CoCM, patients work with a behavioral health consultant (BHC). BHCs are highly trained therapists, either PhD-level clinical psychologists or Licensed Masters-level Social Workers (LMSWs). BHCs are trained to recognize behavioral health concerns, provide short-term therapy, and facilitate connection to relevant community resources. Within CoCM, a BHC and a psychiatrist regularly meet to ensure all individuals receive referrals to community resources and other ancillary services that could support their mental wellbeing. The BHC then communicates back to the outpatient office about the recommendations from the psychiatrist. This model emphasizes the importance of communication between the healthcare provider, the outpatient clinic, and the BHC.

There are demonstrated benefits of the CoCM within the obstetrics setting in improving depression symptoms [24] and engagement in care programs [25]. To date, only one program highlights the importance of maternal mental health in supporting child development; however, this program appears to be in early implementation with no published research at the time of this writing [26].

## 2. Materials and Methods

### 2.1. Study Design

This manuscript reports evaluative aggregate data collected from 7/2021 to 12/2022 (18 months) for enrolled participants who consented to receive clinical care and provided baseline intake metrics.

### 2.2. Setting

MC3 Perinatal is funded by the Michigan Department of Health and Human Services and is housed at Michigan Medicine in the Department of Psychiatry. It combines the CoCM framework (“care” arm) with a Perinatal Psychiatry Consultation program (“consultation” arm).

### 2.3. Participants

Participants who are eligible to receive BHC care through the MC3 Perinatal care arm are 18 years and older, currently pregnant, or postpartum within 12 months of birthing, and are not actively suicidal or homicidal and requiring emergency care. If an eligible patient connects successfully with the BHC, they electronically complete an informed consent for care and a baseline intake and are at that time counted as enrolled in the program.

### 2.4. Program

The consultation arm offers free-of-charge, same-day consultation services to obstetrical providers across the entire state of Michigan (50 counties) and was originally established in 2014. Providers can access same-day consultation to discuss any topics of concern related to mental health as they are treating perinatal patients in their offices (e.g., which medications to select, therapy options available in the region, diagnostic clarifications based on presenting symptoms, etc.). Enrollment is simple, and access to the consultation service is available during weekdays either by phone call or an online consult form; upon review of the request by a care coordinator, the consultation request can either be answered by the coordinator (e.g., regional resources), or the consulting psychiatrist-of-the day, who responds via a direct phone conversation to the provider’s medical questions. Each consultation ends with a written summary of recommendations that are shared with the provider via a secure email.

**Figure 1:**
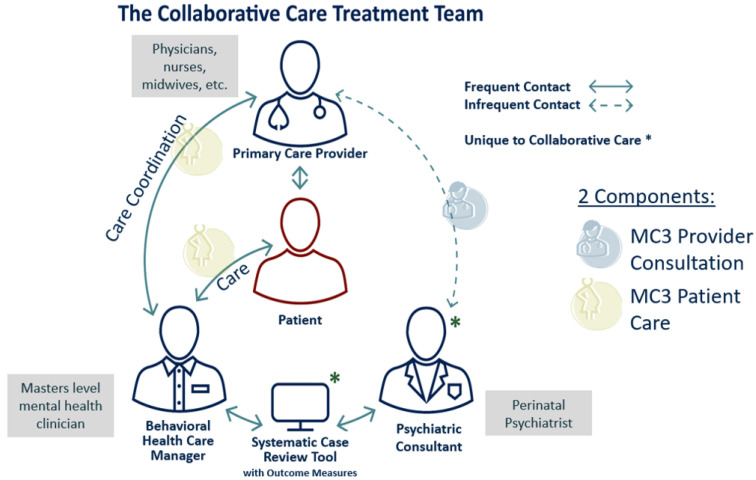
Graphic depiction of the Michigan Clinical Consultation and Care in Perinatal (MC3 Perinatal) Model. Note: * symbolizes that model uses a panel review tool to systematically discuss all patients seen by BHC clinician with a perinatal psychiatrist in weekly sessions.

In response to the critical need for perinatal psychotherapy across Michigan, a direct “care” arm pilot was added in 2021, presently serving 6 counties (with the intent to fully expand across Michigan over time). The care arm enables instant access to virtual one-on-one psychotherapy services for perinatal participants and is grounded in the foundation of CoCM. The full MC3 Perinatal model is depicted in Figure 1.

The consultation arm of MC3 Perinatal resembles other established Perinatal Psychiatry Consultation programs across the US; for example, the MCPAP for Moms program in Massachusetts [23]. Overall, there are currently 26 states with perinatal consultation access programs, and all of them are part of a National Network of Perinatal Psychiatry Access Programs under an umbrella called Lifeline for Moms [27]. We have reported on the consultation program elsewhere [27]. However, the coupling of this consultation service for providers with a direct patient-facing care model is an evidence-based enhancement of our Michigan program. We have not yet reported on this care model, nor presented data. Thus, in this paper, we will elaborate on the clinical workings of the “care” arm, the MC3 Perinatal Care model, and present our pilot implementation data since the model’s inception in 2021.

The goal of the MC3 Perinatal Care arm is to connect pregnant individuals presenting to obstetrical care to licensed BHCs who will initiate interpersonal connection, conduct a mental health evaluation, and promptly start (if indicated) a brief one-on-one therapy process. Participants either self-refer in response to flyers in their OB/GYN’s waiting room or are encouraged by their providers to connect with program; in both cases, engagement is user-friendly using a QR code that directly links the participant to a secure intake webpage. Once engaged, participants automatically connect to the BHC, who initiates telephone or text connection within 48 h (“outreach”) and schedules an initial intake interview at the participant’s convenience. At initial intake, clinicians verify that participants meet eligibility criteria, and if eligible, can enroll by signing an electronic consent. Enrolled participants then provide intake assessments.

In accordance with the CoCM, all enrolled participants are entered into a participant registry after the intake evaluation and are reviewed in the weekly panel meeting with the perinatal psychiatrist. Each participant is discussed at least once at intake and again if the clinical presentation is not improving, as measured objectively by participant-reported monthly metrics for depression and anxiety. During panel review, a 90 min group meeting with one perinatal psychiatrist staffing 3 BHCs, the team collaboratively determines medication, therapy, and resource needs. If medications are recommended, the BHCs coordinate with the obstetrical provider to initiate the prescription of psychotropic medicine. If OB/GYN providers have concerns or questions, they can consult via telephone with the perinatal psychiatrist, using the MC3 Perinatal Consult arm same day.

All participants in MC3 Care are standardly offered up to 8 sessions of therapy with the BHCs. Participants who have longer-term therapy needs are connected through a “warm hand-off” to community therapy resources—for example, home visiting programs. Participants who are hard to refer to community resources are bridged until they can be connected, even if they require more than 8 sessions. The BHCs are trained in several evidence-based interventions, including Cognitive Behavioral Therapy, Dialectical Behavioral Therapy, Acceptance and Commitment Therapy, Interpersonal Therapy, Motivational Interviewing, and Infant Mental Health [28,29,30,31,32,33]. In addition to therapy, BHCs are also trained to provide case management and resourcing to meet tangible needs (e.g., housing, food, diapers, etc.), and undergo training in culturally responsive and trauma-informed care. Given that this program offers a range of services, the program utilizes clinician judgment along with team consultation in the panel review to determine the level and types of services that would benefit the patient (e.g., services may include a medication recommendation for a person that is presenting with severe symptoms of depression or providing psychoeducation for individuals with mild depression). The service level will vary depending on each patient’s presenting needs.

All participants provide monthly self-ratings on depression and anxiety measures (PHQ-9 and GAD-7) until 12 months postpartum, even after they complete their brief therapy course and are referred to the community. These self-rating metrics determine ongoing wellbeing and response to initial treatment. With participant consent, the results of the monthly metrics are also shared with the providers (either the obstetric provider or primary care provider). If a participant is not showing a decrease in symptom scores on the metrics, the case is re-presented at panel review and additional care recommendations are implemented and shared with the providers.

The MC3 Perinatal Care arm is free of charge and delivered to participants fully remote via phone, text messaging, video chat, or email, based on participant preference and ability, at a pace that aligns with participant needs and wishes. Some participants choose weekly therapy/counseling sessions, and some want to space sessions more or less frequently. Session length is individually adjusted based on participant need and preference. Participants are empowered to co-create the care they receive, and as such, the program is culturally responsive, combats stigma, and provides equitable care to vulnerable perinatal populations. Cultural responsiveness is achieved by maintaining a diverse staff from many racial and ethnic backgrounds that is highly experienced in providing perinatal care to urban and rural populations alike, through the provision of paid release time from work and valuing staff’s engagement with staff retreats and self-paced learning around diversity, equity, and inclusion. To promote wellness among the MC3 BHC team, BHCs regularly receive training and consultation. Training includes case conceptualization and the implementation of evidence-based practices, as stated previously [28,29,30,31,32,33]. BHCs are encouraged to select training that fits with their professional development goals and may align with the service needs of the patients they are supporting. Additionally, they engage in reflective consultation, which has been identified as a mechanism to reduce burnout and promote provider wellness, particularly among individuals supporting families with young children [34]. MC3 BHC team members receive reflective consultation biweekly. Currently, the MC3 Perinatal Care arm staff is comprised of three part-time perinatal psychiatrists covering panel reviews and consults, six full or part-time BHCs, and an administrative team for evaluation, outreach, and training activities.

### 2.5. Data Collection

Participant Descriptors: At intake, participants self-reported subjective presenting problem, age, race, and type of insurance (which was used as a proxy for socio-economic status, SES). Insurance status has been utilized as a measure of risk in prior studies [35,36]. Within the United States of America, individuals select insurance via their employer or via the Affordable Care Act [37]. These types of insurance are typically considered private insurance [37]. The US national government sets standards for states to follow and implement regarding who may be eligible for Medicaid benefits [37]. To qualify for Medicaid in the state of Michigan, individuals must be below 133% of the federal poverty level (about USD 18,000 for a single person or USD 37,000 for a family of four) [37]. If a participant had Medicaid insurance, we considered this public insurance [37]. This was used as a proxy for low income [37].

Program Descriptors: All BHC outreach sessions/attempts to reach participants were documented, including categorical time spent on outreach (0–15 min, 16–30 min, 31–45 min, 46–60 min, and 61–75 min), method of outreach (phone, text, email, or virtual), whether a response was received from the participant, and any miscellaneous additional comments. We computed the total number of outreach attempts, total number of times a clinician outreach resulted in a response from the participant, and the total time spent on outreach in two ways: (1) as the numerical average of the categorical ranges, summed across all outreaches and individuals, and (2) as the percent of outreaches conducted in each category of time. BHC therapy sessions were documented in several ways: using surveys, BHCs documented for each participant (1) the time spent in a session (0–15 min, 16–30 min, 31–45 min, 46–60 min, and 61–75 min); (2) which metrics were used in a particular session; and (3) which interventions were utilized during a session; finally, by using a participant tracking tool, BHCs indicated (4) the date on which a session was scheduled, missed, or successfully completed. The total number of sessions was computed as the total number of successfully completed sessions. The total time spent in sessions was computed as the numerical average of the categorical ranges of time, summed across all sessions and individuals. Participant satisfaction was completed at baseline and once a month for each month the participant was enrolled in services. This measure included six items from the Client Satisfaction Questionnaire (CSQ-8), which determined participant levels of satisfaction on a 4-point scale, from “Strongly Disagree” to “Strongly Agree” [38]. For this paper, the “Strongly Agree” and “Agree” rating categories were combined and the “Disagree” and “Strongly Disagree” rating categories were combined. This measure also included three open-ended questions assessing what the participant felt was most and least useful about the program, and what they would change to make the program better. Response themes from open-ended satisfaction questions were coded and verified by the study team. The Satisfaction surveys were not implemented until July 2022; therefore, this paper reflects data collected from July 2022 to February 2023.

Mental Health Variables: The 5Ps Prenatal Substance Abuse Screen: We assessed for risk of substance abuse at baseline with the 5Ps Prenatal Substance Abuse Screen [39]. This measure includes questions about whether the participant’s parents, friends, or partners have had problems with alcohol or drug use, and whether the participant has had problems with alcohol or drug use prior to pregnancy. An endorsement of any of the 5Ps items was used to indicate risk of substance use. Starting approximately midway through the data collection period, the NIDA Quick screen questionnaire was introduced [40], which asked participants to report on use of tobacco, marijuana, cocaine, opioids, stimulants and crystal meth.

Interpersonal Violence Risk Screen: We assessed for risk of interpersonal violence (IPV) at baseline with 4 items created by the study team. This measure asks, in the last year, whether you have been: “afraid of someone close (or less close) to you?”, “hit, slapped, kicked, pushed, shoved, or otherwise physically hurt by someone close (or less close) to you?”, “frequently made upset, ashamed, or embarrassed by someone close (or less close) to you?”, and “forced to have sex by someone close (or less close) to you?”. Endorsement of one or more items was flagged as risk of IPV. If a person indicated they were experiencing IPV risk, the clinicians identified this in reviewing the data, and followed a pre-defined risk management protocol to address these concerns with the patient. If the patient was at imminent risk, the BHC provided instant case management and care coordination to connect the patient with domestic violence shelters and other IPV resources.

The Generalized Anxiety Disorder-7 (GAD-7), a 7-item questionnaire, was used to assess symptoms of generalized anxiety disorder (GAD) [41,42]. Participants answered questions on a 4-point Likert scale (0 = “Not at all” to 3 = “Nearly every day”) indicating how often they have been bothered by problems; for example: “Not being able to stop or control worrying”, and “Becoming easily annoyed or irritable”. Scores were totaled to suggest the current severity of symptoms: <4 = minimal anxiety, 5–9 mild anxiety, 10–14 moderate anxiety, and 15–21 = severe anxiety. The GAD-7 has been delivered and validated among pregnant individuals with a cut-off score of 7 [42]. This measure was completed at baseline, as well as once per month for each month the participant was enrolled in services.

The Patient Health Questionnaire (PHQ) is a 9-item self-report measure used to assess symptoms of major depressive disorder [43,44]. Participants answer questions on a 4-point Likert type scale (0 = “Not at all” to 3 = “Nearly every day”) indicating how often they have been bothered by problems, for example: “Feeling down, depressed, or hopeless”, and “Trouble concentrating on things”. Scores were then totaled to suggest current severity of symptoms. The clinical cutoff of 10 was used to differentiate probable depression diagnosis. The PHQ was selected over the Edinburg Postnatal Depression Scale as it has been found to have similar sensitivity [45]. The PHQ-9 has been validated with pregnant and postpartum individuals [46,47]. This measure was completed at baseline and once a month for each month the participant was enrolled in services. The measures were completed with or without a BHC. Safety protocols are implemented to address endorsements of suicidality on the PHQ-9. If the item was endorsed in the presence of a BHC, the BHC completed a predefined risk management protocol identifying risk level for the patient and connected the patient to appropriate resources. If the patient completed the PHQ-9, without the BHC present, the system in which the data are collected prompted the patient with safety and suicide prevention resources. Clinicians reviewed the measures daily, and when the clinician identified a patient as at risk based on the PHQ-9, the clinician completed the pre-defined risk management protocol to address suicidal ideation with the patient and connect the patient to appropriate resources.

### 2.6. Data Analysis

We provide descriptive statistics of the participants presenting for MC3 Perinatal Care arm services, including their presenting concerns, and levels of mental health symptoms from the time of recruitment into services. Survival analysis, using Cox regression (SAS PROC PHREG), was used to examine retention in services; we report hazard ratios for this analysis. We examined Black race as a predictor of retention; we did not have a large enough sample of participants identifying as other races or Latino/a to include as predictors. SAS PROC LIFETEST was used to plot the survival trajectories. We used logistic regression to examine demographic predictors of enrolling for those who were referred.

To examine the change in mental health symptoms over time, we first examined selective attrition by using logistic regression to predict missingness. For each time point, we tested anxiety and depression symptoms at the prior time point as predictors, using logistic regression. Next, trajectories of mental health symptoms were modeled using latent growth modeling in Mplus, using full information maximum likelihood to handle missing data. Data analyses were performed using SAS 9.4 and Mplus v. 8.8

### 2.7. Ethical Considerations

This project was approved as a Quality Improvement, Quality Assurance project within the University of Michigan by our Institutional Review Board (IRB).

## 3. Results

### 3.1. Participant and Program Descriptors

Since the program’s inception in July of 2021, 285 participants have been referred to the MC3 Perinatal Care arm. The race/ethnicity distribution of the referred participants is shown in Table 1. Of the 285 participants referred, 66% endorsed White race, 30% Black race, and 2% Asian race; less than 6% identified as Hispanic or Latino. In total, 47% were enrolled in public insurance (2% were uninsured), and the mean age was 29.9 years (SD = 5.5 years). At the time of referral, 49% were pregnant and 51% were postpartum. Of all those referred, we engaged 259 participants (91%) and enrolled 209 (72%). Those enrolled were more likely to have private insurance (OR = 2.5, *p* = 0.0027), endorse White race (OR = 2.07, *p* = 0.013), and be older (OR = 1.17, *p* = 0.0093); however, pregnancy status did not predict enrollment (OR for being pregnant vs. postpartum = 0.81, *p* = 0.44).

Over a third of all enrolled participants (n = 63) reported substance use risk using the 5Ps Prenatal Substance Abuse Screen [39]. This screen includes past problems with substance use as well as partner and family substance use problems. Current use of substances was endorsed by 26.2% of women using the NIDA Screen [40] on a subsample of n = 99. When probed on the type of substances used, 11.1% endorsed the use of tobacco (in the last month, 3.0% endorsed its use once or twice, and 8.1% endorsed its use daily or almost daily), 15.1% endorsed the use of marijuana (in the last month, 7.1% endorsed its use once or twice, 2.0% endorsed its use weekly, and 6.1% endorsed its use daily or almost daily) and no participants endorsed the use of cocaine, opioids, stimulants or crystal meth.

The majority of participants indicated a presenting problem that led them to seek clinical support for anxiety (65%) and depression (51%), followed closely by general and parenting stress (43%). Other presenting concerns included breastfeeding, high-risk pregnancy or miscarriage, trauma and post-traumatic stress symptoms, panic attacks, need for parenting and general life support and resources, wish for medications, and mood challenges. Self-reported scores on validated measures of depression (PHQ-9) and anxiety (GAD-7) confirmed high levels of clinical depression (53%) and anxiety (60–76%) at intake. Sixteen percent of the enrolled participants (n = 28) endorsed at least one risk item for IPV.

#### 3.1.1. Outreach and Treatment Sessions

Table 2 describes the outreach by clinicians to the referred participants. The target was to have outreach within 24 h of the referral. Of the total of 285 referrals, we reached this target for 243, or 81% of referrals. In 51—or 17% of cases—there was a delay from 2 to 6 days, and in 5—or 2% of cases—there was a delay greater than 6 days. In 2—or 1% of cases—we were missing such data.

A total of 209 participants enrolled in the treatment sessions (72%). Enrolled participants received 1 to 25 sessions, with an average of 6.6 (SD = 4.5) sessions. Clinicians conducted 1386 sessions for a total of 845 h in session with an average of 4.9 (SD = 3.9) hours in session per participant.

#### 3.1.2. Participant Satisfaction

Overall, satisfaction with the MC3 Perinatal Care service was high, with 97% of individuals endorsing satisfaction with the services they received (See Table 3). The aggregate results from open-ended questions (not shown in table) suggested the most useful parts of the program were the support or therapy they received (endorsed by 71% of participants), along with the resources provided—including medication management (46%). Also noted as useful parts of the program were care coordination (13%), the flexibility of the BHCs, and zero program costs (21%). When asked what their least favorite parts of the program were, most participants (86%) indicated they found it all useful, while some indicated difficulty finding referrals to outside services (9%) and that there were not enough visits with BHCs (5%). When asked what they would change to make the program better, most participants indicated they were satisfied with the program as it is (73%), with some indicating they would like the program to be more well known by the public (7%) and the ability to include spouses in the services (7%).

#### 3.1.3. Retention in Treatment

**Figure 2:**
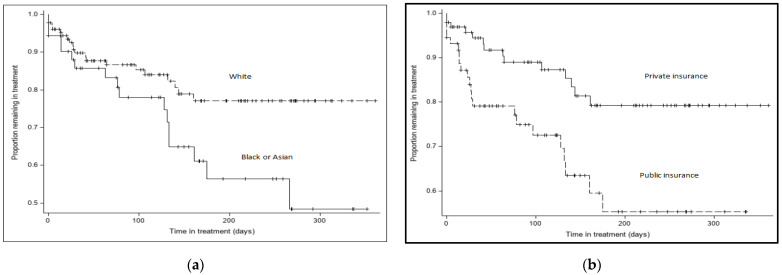
Plots of survival to termination of treatment separated by (**a**) race; (**b**) type of insurance.

To understand how the program retained a diverse group of participants, we explored demographic factors that predicted retention in treatment. For the 209 enrolled participants, we examined baseline predictors of remaining in treatment, including Black race, type of insurance, IPV risk, and substance use risk. (HR). Black race (HR = 2.42, *p* = 0.007) and public insurance (HR = 2.54, *p* = 0.007) predicted faster attrition from treatment; IPV risk (HR = 0.58, *p* = 0.46) and substance use risk (HR= 1.40, *p* = 0.44) were not related to attrition rate. Figure 2 shows plots of survival to the termination of treatment separated by (a) Black race and (b) type of insurance. Those who endorsed White as race were more likely to remain in treatment longer (Figure 2a). From the beginning of treatment, there was less attrition in the White group, and the differences increased over time. At six months following baseline, 77% of those who endorsed White were still engaged in treatment, whereas only 56% of those who did not endorse White were still engaged. Similarly, those who reported having public insurance or no insurance were likely to leave treatment before those who reported having private insurance (Figure 2b). These differences are substantial early in treatment. By one month postbaseline, 94% of those with private insurance remained in treatment, whereas only 79% of those with public insurance remained.

### 3.2. Mental Health Outcomes

**Figure 3:**
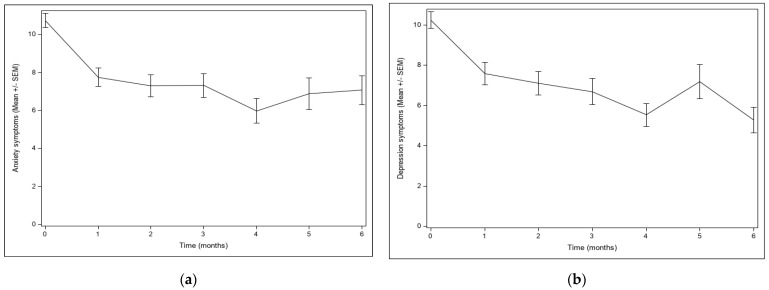
Mental health symptoms over time: (**a**) anxiety symptoms over time (mean and standard deviation); (**b**) depression symptoms over time (mean and standard deviation).

Trajectories of anxiety and depression symptoms were modeled for the 209 enrolled participants using latent growth modeling. We first tested whether there was selective attrition based on anxiety and depression symptoms. At each time point, starting with the second month, we found that anxiety and depression from the previous time point did not predict missingness. Next, for anxiety and depression symptoms separately, we fit models for the baseline and for each of the first six months of follow-up assessments, for which there were sufficient data to avoid convergence problems in estimation. For both anxiety and depression symptoms, cubic models had better fit than linear or quadratic models. As seen in Figure 3, the mean levels of symptoms for both anxiety (Figure 3a) and depression symptoms (Figure 3b) decreased over time. The most dramatic decreases were in the first month, when most of the treatment occurred. The modeling showed significant decreases in anxiety from baseline to month 1 (delta = 2.36, Wald test = 46.96, *p* < 0.0001), as well as from month 1 to month 6 (delta= 1.96, Wald test = 7.94, *p* = 0.0048) and in depression from baseline to month 1 (delta = 2.30, Wald test = 53.42, *p* < 0.0001) and from month 1 to month 6 (delta = 1.94, Wald test = 14.46, *p* = 0.0001).

## 4. Discussion

In this manuscript, we presented an evidence-based perinatal mental health collaborative care model, MC3 Perinatal, which combines instant access to remote psychotherapy for perinatal patients (“care” arm) with same-day provider consultation on medication questions (“consultation” arm). We presented early implementation data for the care arm, including participant demographics, participant satisfaction, retention, and mental health outcomes.

Of the 285 referred participants in the past 18 months since the pilot began, we connected with 259 participants (91% of those referred) and enrolled 209 into services (72% of those referred). This rate of engagement is comparable to or higher than some similar studies, such as one 2014 study that reported a 56% enrollment rate in a CBT program for perinatal depression [47] or a 2014 Australian study of the implementation of a dedicated perinatal and infant mental health service showing 71% engagement in at-risk perinatal participants [48]. Our finding of 72% engagement suggests comparable or superior receptivity to perinatal mental healthcare in our at-risk perinatal population.

97% of the enrolled individuals endorsed satisfaction with the services they received. The participants felt that the most useful parts of the program were the support or therapy they received (endorsed by 71% of participants), in addition to the resources provided—including medication management (46%). Also noted as useful parts of the program were care coordination and the flexibility of the clinicians and program costs. Most participants (86%) indicated they found all the services useful, an encouraging finding. Improvement suggestions seemed to be relatively easy to incorporate, including ideas such as improved referrals to outside services, increased visit numbers with clinicians, increased public knowledge of the program, and the ability to include spouses in the services. When asked what they would change to make the program better, most participants indicated they were satisfied with the program as it is (73%). These results support the idea that CoCM generally garners high patient satisfaction across specialties, including both patient-reported and provider-perceived satisfaction [49]. A meta-analysis of 167 studies of integrated care found strong evidence for the relationship between integrated care and high patient satisfaction, and a 2022 study of CoCM for low-income racial–ethnic minority groups found similarly high (74%) patient-reported satisfaction [50,51]. Another CoCM intervention for at-risk perinatal populations found rates of self-reported patient satisfaction ranged from 62% at 18 months to 71% at 3 months [52]. Our results suggest a similarly high level of patient satisfaction.

Our outreach data indicate that we successfully reached individuals across the spectrum of race, insurance status, and mental health conditions—including a relatively even split of depression and anxiety—as well as representation of other risk factors, such as IPV (16% of participants) and substance use (34%), and public insurance or uninsured status (~50%). However, we also found that despite our ability to initially engage vulnerable groups including Black participants, we were less successful in the retention of these populations. Black race and public insurance predicted faster attrition from treatment. Non-White participants in general were less engaged with care at six months than White participants: 56% of non-White participants versus 77% of White participants. This finding is commensurate with national data and speaks to the barriers that Black participants may face in staying in mental healthcare [53,54]. The existing literature cites potential barriers for Black participants in access to mental healthcare on multiple levels of society, including broader systemic factors (wait times, lack of access to childcare or transportation, geographical challenges, financial barriers, etc.), practitioner-specific factors (racism and discrimination from staff, a paucity of non-White or Black providers, an inability to offer culturally competent care, etc.), and personal or community-related factors (internalized stigma, cumulative trauma, sociocultural expectations, etc.) [55,56,57]. One Canadian study noted support from family and friends and a good relationship with providers as facilitators to follow through with care [58]. A 2021 study identified a significant increase in patient utilization of urgent telemedicine mental healthcare among Black patients, suggesting that Black patients may be more comfortable accessing this type of mental healthcare [59]. Because the patient-facing MC3 Perinatal care arm is centered around telehealth counseling, it is likely that this fact positively influenced initial engagement with the service. It is important to note that, for Black communities, pregnancy, birth, and the postpartum period are journeys of life with deep spiritual, mental, and emotional connotations [60]. Care that focuses solely on the individual and excludes their families and communities may not be the most optimal way to sustainably engage Black participants [60]. This sentiment is possibly supported by our finding that 7% of MC3 participants commented on their desire to include spouses in services. Our program did not achieve sustained engagement, and future work will need to focus on better understanding how to keep engagement over time and prevent premature attrition.

Historically, substance use disorder (SUD) and IPV are predictors of poor retention in mental health programming, particularly when combined with underlying severe psychopathology, such as severe perinatal anxiety [61,62,63,64,65,66,67]. In our study, because IPV and substance use risk did not predict attrition from the program, this highlights the importance of accessible, patient-facing mental healthcare for vulnerable patient populations. Integrated care is important for these patients, as there is a significant link between experiencing domestic violence and high-level symptoms of perinatal depression, anxiety, and PTSD [64,65,66,68]. In addition, IPV is a risk factor for the suicide of perinatal patients, as a history of IPV has been noted in nearly half of mothers who died by suicide [3]. Substance use is also a factor in perinatal death, as noted in a 2020 study that established a link between perinatal death, substance use, and poor mental healthcare [3]. Critically, a 2021 article commented on the importance of the initial identification of these risk factors, as providers cannot assist patients if they are not aware of their histories [69]. This emphasizes the importance of open communication between OB/GYN providers and BHCs, both in order to identify overall risk and to offer tailored resources and interventions to mitigate catastrophic negative outcomes.

The encouraging outcomes described in this paper highlight the benefit of MC3 Perinatal in vulnerable populations—in this case, persons with a history of IPV and substance abuse—and the progress MC3 Perinatal makes toward the ultimate goal of equitable, accessible care. We are attempting to address attrition from the program by continuing to follow-up with participants. We have recently implemented an extended outreach protocol that stipulates contact for up to one year—or if the participant indicates they are no longer interested in services.

This study is not without limitations. We lacked a comparison group of perinatal participants against whom to objectively evaluate the efficacy of the program. This was not performed because of the challenges presented in obtaining comparison data on a matched group of perinatal participants in counties not yet included in this program. We did not evaluate participants by ethnicity, potentially limiting conclusions in this area. We did not collect the demographics of staff members, though we acknowledge that this information may have better informed the reader’s understanding of the program’s cultural responsiveness. We did not measure certain variables which may be considered important, including inpatient stays for mental health concerns and rates of mortality or morbidity. We only presented data on a small pilot sample, especially in follow-up data on depression and anxiety across the postpartum period, as the program is relatively new and has been only in existence since late 2021. However, enrollment is rising, and data collection is ongoing, which will continue to shape our findings.

## 5. Conclusions

The MC3 Perinatal care program is a collaborative model of care that attempts to increase equitable access to perinatal mental health and facilitate access to outside resources. MC3 Perinatal care aims to step beyond traditional case management to offer highly accessible, short-term, evidence-based psychotherapy and the yearlong monitoring of symptoms beyond the active therapy episode. As such, MC3 Perinatal care hopes to provide holistic and ongoing care that is specifically tailored to perinatal patients’ needs and with a culturally responsive and trauma-informed lens. The model is delivered remotely, based on the modality and frequency that are desired and accepted by the participant, which is thought to increase access and acceptance for the service. While 97% of participants endorsed satisfaction with the services and the program showed benefits to mental health, we also acknowledge ongoing shortcomings as Black race and public insurance predicted faster attrition from treatment. This finding is crucial and can inform areas for opportunity in modifying the program to better serve this target group. Contrary to the literature, risks for interpersonal violence exposure and substance use were not related to attrition. Our program addresses the complexity of serving vulnerable populations by addressing access, acceptability, resourcing, care navigation and coordination and counseling, all provided within the context of a behavioral health and medical team. Our pilot evaluation data show promise upon which to adapt and optimize to enhance the program’s beneficial impact for the community.

## Figures and Tables

**Table 1:** Demographics and Baseline Risks.

	M/%	SD/n
Age	29.92	5.51
Race: White	66.40	163
Black	29.55	74
Asian	2.02	5
American Indian or Alaska Native	0.74	2
Native Hawaiian or Other Pacific Islander	0.74	2
Ethnicity: Hispanic or Latino	5.44	13
Insurance: Public	47.23	110
Private	50.64	120
Uninsured	2.13	5
Depression *	52.86	111
Anxiety * (score of 7+)	76.08	159
Anxiety * (score of 10+)	59.81	125
Interpersonal Violence Risk	15.91	28
Substance Use Risk	35.00	63

* Percent meeting cutoff at baseline.

**Table 2:** Program Delivery.

Outreach	M (SD)/% (n)
Days between referral and first outreach attempt	1.54 (8.03)
Outreach attempts before contact	2.09 (1.55)
Outreach attempts	
Texts	64.44% (2197)
Calls	18.22% (622)
Emails	16.90% (577)
Video calls	0.05% (18)
Outreaches resulting in response	44.76% (1362)
Outreach time (min)	
0–15	93.08% (2826)
16–30	5.50% (167)
31–45	0.86% (26)
46–60	0.16% (5)

**Table 3:** Patient Satisfaction Survey (n = 32).

	Disagree	Agree
I am satisfied with the services that I received.	3%	97%
This program lived up to my expectations.	3%	97%
This program met my needs.	6%	94%
I would recommend this program to a friend if they were in need of similar help.	3%	97%
If I were to seek help again, I would come back to this program.	3%	97%
This program has helped me feel better equipped to handle the demands of this period in my life.	6%	94%

## Data Availability

The data are available on request due to restrictions (privacy and ethical). The deidentified data presented in this study are available on request from the corresponding author. The data are not publicly available due to clinical privacy restrictions.
